# Mass Spectrometry Imaging for Reliable and Fast Classification of Non-Small Cell Lung Cancer Subtypes

**DOI:** 10.3390/cancers12092704

**Published:** 2020-09-21

**Authors:** Mark Kriegsmann, Christiane Zgorzelski, Rita Casadonte, Kristina Schwamborn, Thomas Muley, Hauke Winter, Martin Eichhorn, Florian Eichhorn, Arne Warth, Soeren-Oliver Deininger, Petros Christopoulos, Michael Thomas, Thomas Longerich, Albrecht Stenzinger, Wilko Weichert, Carsten Müller-Tidow, Jörg Kriegsmann, Peter Schirmacher, Katharina Kriegsmann

**Affiliations:** 1 Institute of Pathology, University of Heidelberg, 69120 Heidelberg, Germany; christiane.zgorzelski@med.uni-heidelberg.de (C.Z.); thomas.longerich@med.uni-heidelberg.de (T.L.); albrecht.stenzinger@med.uni-heidelberg.de (A.S.); peter.schirmacher@med.uni-heidelberg.de (P.S.); 2 Translational Lung Research Centre Heidelberg, Member of the German Centre for Lung Research (DZL), 69120 Heidelberg, Germany; thomas.muley@med.uni-heidelberg.de (T.M.); hauke.winter@med.uni-heidelberg.de (H.W.); martin.eichhorn@med.uni-heidelberg.de (M.E.); florian.eichhorn@med.uni-heidelberg.de (F.E.); petros.christopoulos@med.uni-heidelberg.de (P.C.); michael.thomas@med.uni-heidelberg.de (M.T.); 3 Proteopath Trier, 54296 Trier, Germany; r.casadonte@molekularpathologie-trier.de (R.C.); kriegsmann@patho-trier.de (J.K.); 4 Institute of Pathology, TU Munich, 81675 Munich, Germany; kschwamborn@tum.de (K.S.); wilko.weichert@tum.de (W.W.); 5 Department of Thoracic Surgery, Thoraxklinik at Heidelberg University, 69120 Heidelberg, Germany; 6 Institute of Pathology, Cytopathology, and Molecular Pathology, UEGP Gießen/Wetzlar/Limburg, 35578 Wetzlar, Germany; warth@patho-uegp.de; 7 Bruker Daltonik, 28359 Bremen, Germany; soeren.deininger@bruker.com; 8 Department of Thoracic Oncology, Thoraxklinik at Heidelberg University, 69120 Heidelberg, Germany; 9 German Cancer Consortium (DKTK)-German Cancer Research Center (DKFZ), 69120 Heidelberg, Germany; 10 Department of Hematology, Oncology and Rheumatology, University of Heidelberg, 69120 Heidelberg, Germany; carsten.mueller-tidow@med.uni-heidelberg.de; 11 Center for Histology, Cytology and Molecular Diagnostic Trier, 54296 Trier, Germany; 12 Institute for Molecular Pathology Trier, 54296 Trier, Germany; 13 Danube Private University, 3500 Krems, Austria

**Keywords:** mass spectrometry imaging, mass spectrometry, NSCLC, lung cancer

## Abstract

**Simple Summary:**

Diagnostic subtyping of non-small cell lung cancer is paramount for therapy stratification. Our study shows that the subtyping into pulmonary adenocarcinoma and pulmonary squamous cell carcinoma by mass spectrometry imaging is rapid and accurate using limited tissue material.

**Abstract:**

Subtyping of non-small cell lung cancer (NSCLC) is paramount for therapy stratification. In this study, we analyzed the largest NSCLC cohort by mass spectrometry imaging (MSI) to date. We sought to test different classification algorithms and to validate results obtained in smaller patient cohorts. Tissue microarrays (TMAs) from including adenocarcinoma (ADC, *n* = 499) and squamous cell carcinoma (SqCC, *n* = 440), were analyzed. Linear discriminant analysis, support vector machine, and random forest (RF) were applied using samples randomly assigned for training (66%) and validation (33%). The *m/z* species most relevant for the classification were identified by on-tissue tandem mass spectrometry and validated by immunohistochemistry (IHC). Measurements from multiple TMAs were comparable using standardized protocols. RF yielded the best classification results. The classification accuracy decreased after including less than six of the most relevant *m/z* species. The sensitivity and specificity of MSI in the validation cohort were 92.9% and 89.3%, comparable to IHC. The most important protein for the discrimination of both tumors was cytokeratin 5. We investigated the largest NSCLC cohort by MSI to date and found that the classification of NSCLC into ADC and SqCC is possible with high accuracy using a limited set of *m/z* species.

## 1. Introduction

Lung cancer, which is histologically classified as non-small cell lung cancer (NSCLC, 85%) and small cell lung cancer (15%), is the most common cancer in men and the second most common in women worldwide [1]. Despite recent advances in targeted therapy, 35–40% of patients are diagnosed at an advanced clinical stage with a 5-year survival rate of approximately 19% [1].

Treatment is based on the histological subtype and genetic alterations [2]. The main histological subtypes within the NSCLC group are adenocarcinoma (ADC) and squamous cell carcinoma (SqCC). At an advanced clinical stage, individualized therapy highly depends on genetic aberrations involving various genes [3]. Recently, the introduction of immune checkpoint and kinase inhibitors has improved prognosis for patients without genetic alterations in these target genes [4,5].

In this context, correct diagnosis of ADC and SqCC is paramount because some genetic alterations are mainly restricted to (and tested in) non-SqCC, and some therapies—such as treatment with bevacizumab—are contraindicated in SqCC.

In well-to-moderately differentiated tumors, the distinction between ADC and SqCC is often possible based on morphology alone. In poorly differentiated tumors, adjunct immunohistochemical (IHC) staining may be necessary. Cytokeratin (CK) 5/6 and p40 are recommended markers to identify squamous lineage, and Napsin-A and thyroid transcription factor 1 (TTF-1) are commonly used markers for ADC. To save tissue for subsequent molecular analyses, parallel application of TTF-1 and p40 as the most reliable marker combination is recommended [6,7]. A less extensive workup will result in decreased diagnostic precision, which is suboptimal [8].

To tackle this problem, we previously used mass spectrometry imaging (MSI), which relies on the analysis of a single tissue section, and created a linear discriminant analysis (LDA) model to reliably distinguish ADC and SqCC. We tested this algorithm on 118 separate patient samples, including 58 ADC and 60 SqCC samples, and were able to discern both entities with high accuracy [9].

In the current study, we expanded the data acquired in the previous investigation by performing the following: (i) upscaling the cohort to a total of 939 NSCLC formalin-fixed paraffin-embedded (FFPE) tissue samples assembled on tissue microarrays (TMAs); (ii) using a novel high-speed mass spectrometry instrument that reduces the MSI analysis time to less than one hour; (iii) developing an MSI data analysis workflow to reveal markers that are most relevant to the classification; and (iv) comparing different machine learning algorithms and accuracy rates of MSI data with the current IHC gold standard.

## 2. Results

### 2.1. Patient Characteristics

Overall, 939 patients diagnosed with either ADC (*n* = 499) or SqCC (*n* = 440) of the lung were analyzed. In the ADC cohort, the male/female ratio was 1.4. Median age was 63 (30–89) years. Most patients were diagnosed either in stage I (*n* = 201, 40%), II (*n* = 101, 20%) or III (*n* = 173, 35%), and only 5% of the patients (*n* = 24) had stage IV disease at diagnosis. The majority of SqCC patients were male, with a male/female ratio of 5.3 in the SqCC cohort. Median age at diagnosis was 65 (38–83) years. Similar to ADC, only a small proportion of patients were diagnosed with stage IV disease (*n* = 5, 1%), and stages I, II, and III accounted for 149 (34%), 171 (39%), and 115 (26%) patients, respectively. Patient characteristics are summarized in Table 1.

### 2.2. Non-Small Cell Lung Cancer Classification Based on Mass Spectrometry Imaging Data

**Figure 1:**
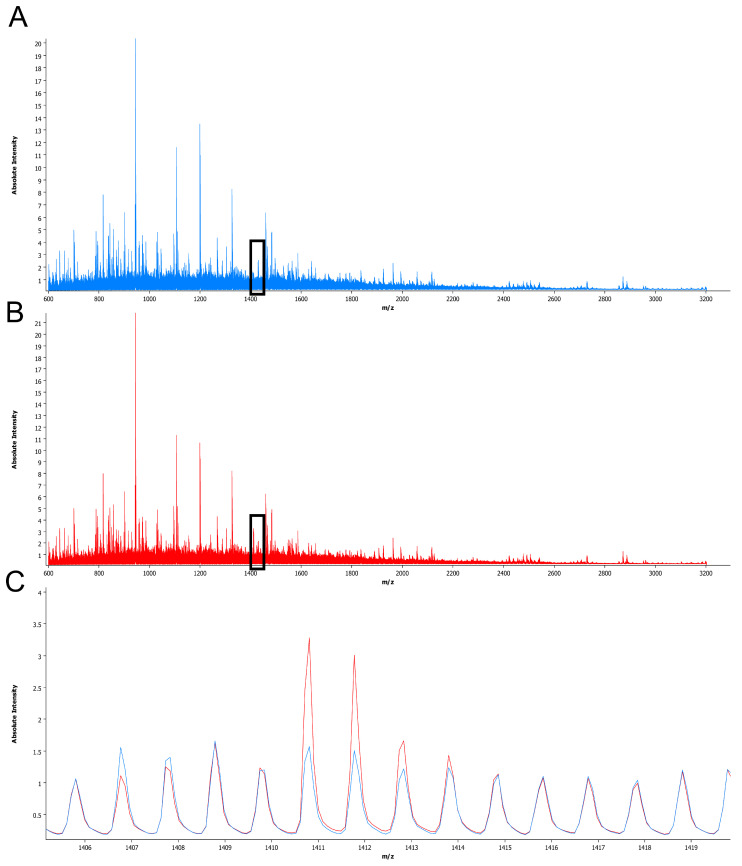
Sum spectra obtained from adenocarcinoma and squamous cell carcinoma samples. The mean spectra from adenocarcinomas are displayed in blue (**A**) and those from squamous cell carcinomas are displayed in red (**B**). A magnified view of the range of *m/z* 1406–1419 (**C**), black triangle in (**A**) and (**B**) reveals peaks with different intensities between both entities, e.g., at *m/z* 1410.7.

Upon MSI analysis, sum spectra were generated over all analyzed samples to select *m/z* values for further data evaluation (Figure 1). In the initial selection, 263 *m/z* values within the 602.4–3184.5 *m/z* range were identified. Sixty-eight of these *m/z* values showed a peak intensity below 1 and were excluded to ensure high quality of the MSI signal for further data analysis. Therefore, further evaluation was performed on the remaining 195 *m/z* values within the 602.4–2728.2 *m/z* range. The intensity of the selected *m/z* values ranged between 1.00 (predefined) and 18.47.

**Figure 2:**
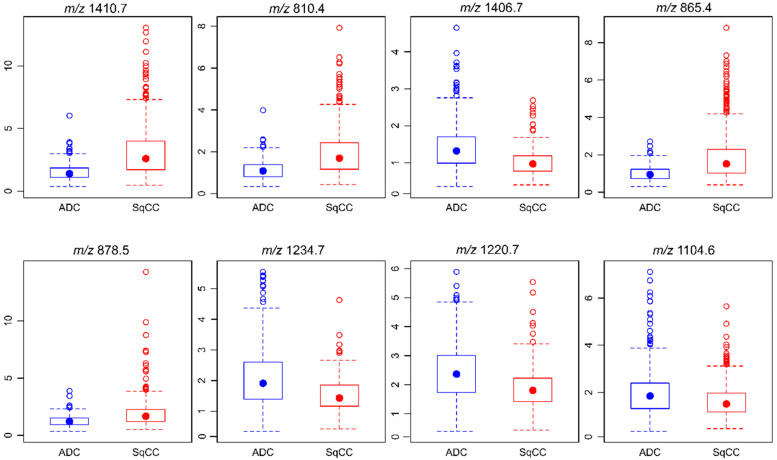
Intensity of *m/z* values contributing to the classification model regarding the entity. The eight *m/z* values that contribute most to the random forest model classification of ADC and SqCC are shown. Peak intensities are presented as box plots with regard to the entity. In particular, the median demonstrates the differences in *m/z* values between the two entities.

Aiming to achieve a correct tumor classification based on MSI data, three established regression and classification algorithms (i.e., Random forest (RF), support vector machine (SVM), and linear discriminant analysis (LDA)) were selected for initial classification training (training set, two-thirds of all cases) and subsequent validation (validation set, one-third of all cases). As the highest classification accuracy was obtained with RF (0.906, CI_95%_ 0.868–0.935) compared with SVM (0.871, CI_95%_ 0.829–0.906) and LDA (0.827, CI_95%_ 0.781–0.867), the RF algorithm was selected for further analyses (Figure S1A). The 8 *m/z* values that contributed most to the RF model classifying ADC and SqCC samples were selected. In descending order of importance, those were *m/z* 1410.7, 810.4, 1406.7, 865.4, 878.5, 1234.7, 1220.7, and 1104.6 (Figure S1B). The median intensity values detected in ADC and SqCC were *m/z* 1410.7—ADC 1.39 and SqCC 2.59, m/z 810.4—ADC 1.08 and SqCC 1.69, m/z 1406.7—ADC 1.32 and SqCC 0.97, *m/z* 865.4—ADC 0.95 and SqCC 1.52, m/z 878.5—ADC 1.20 and SqCC 1.67, *m/z* 1234.7—ADC 1.91 and SqCC 1.43, *m/z* 1220.7—ADC 2.37 and SqCC 1.81, and *m/z* 1104.6—ADC 1.85 and SqCC 1.51. Compared to that in ADC, *m/z* 1410.7, 810.4, 865.4, and 878.5 showed higher intensity values in SqCC. In contrast, *m/z* 1406.7, 1234.7, 1220.7, and 1104.6 displayed higher intensities in ADC than in SqCC (Figure 2).

Including the eight most important *m/z* values into the RF classification algorithm resulted in a prediction accuracy of 0.906, a sensitivity of 0.929 and a specificity of 0.879, which were similar to the metrics obtained in an RF model including all (*n* = 195) *m/z* values as variables. To answer the question of whether the RF classification accuracy between ADC and SqCC tumors can be maintained with a reduced set of *m/z* values, the number of included *m/z* values was modified in subsequent RF models. The prediction accuracy was maintained at the level of approximately 0.9 when the number of included *m/z* peaks was reduced from eight to six. However, the inclusion of less than six *m/z* peaks led to a significant decrease in the prediction accuracy, and finally, a classification model based on only one *m/z* value (*m/z* 1410.7) resulted in a poor prediction accuracy of only 0.676 (Table 2).

### 2.3. Plausibility of the Tumor Classification Model Based on the Selected m/z Values

**Figure 3:**
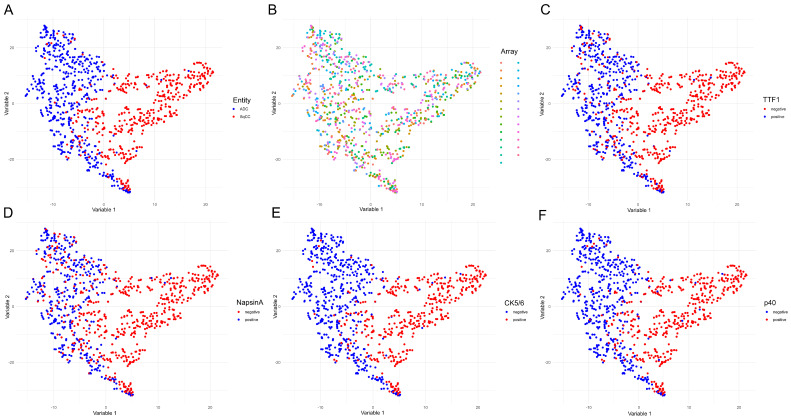
T-distributed stochastic neighbor embedding analysis reveals the plausibility of adenocarcinoma and squamous cell carcinoma classification based on the selected *m/z* values. T-distributed stochastic neighbor embedding analysis was performed including the eight previously selected *m/z* peaks (*m/z* 1410.7, 810.4, 1406.7, 865.4, 878.5, 1234.7, 1220.7, and 1104.6). Each dot represents a single case. The data are presented with regard to tumor entity (**A**); analyzed TMA (each color represents a TMA (tissue microarray), with 24 TMAs total (**B**); TTF1 (thyroid transcription factor 1) (**C**); Napsin-A (**D**); CK5/6 (cytokeratin 5/6) (**E**); and p40 (**F**).

To show that the tumor classification model based on the eight selected *m/z* values (i.e., *m/z* 1410.7, 810.4, 1406.7, 865.4, 878.5, 1234.7, 1220.7, and 1104.6) is plausible, a T-distributed stochastic neighbor embedding (t-SNE) visualization was performed. Therefore, it was demonstrated that ADC and SqCC can be separated by the *m/z* intensity profiles of the eight selected peaks (Figure 3A). Moreover, we found no clusters that indicated separation by TMA (Figure 3B), demonstrating that the *m/z* peak intensities obtained from different TMAs were within a similar range. Most importantly, the ADC and SqCC clusters obtained based on the intensities of the eight selected peaks corresponded to the expected immunohistochemical profile. Specifically, the t-SNE separation of ADC and SqCC corresponded either to the ADC immunohistochemical profile (TTF-1 positive, Napsin positive, CK5/6 negative, and p40 negative) or to the SqCC immunohistochemical profile (TTF-1 negative, Napsin negative, CK5/6 positive, and p40 positive; Figure 3C–F).

### 2.4. Identification of Diagnostically Relevant m/z Values and Immunohistochemical Validation

Out of the eight selected *m/z* values, we were able to identify four by MS/MS. The results from the MS/MS identification are depicted in Table S1. Two peaks could not be identified due to a limited peak signal but could tentatively be identified when comparing results with other studies investigating human tissue samples. One *m/z* value was not identified. Six identified peptide peaks were derived from CK species. Three peptide peaks were identified as fragments from CK5 and type II (*m/z* 1410.7, 810.4, and 865.4). One peptide peak was identified as CK7, type II (*m/z* 1104.6) [10]. One peptide peak was classified as collagen alpha 2(I) chain (*m/z* 1234.7) [11]. For *m/z* 1406.7, two possible peptide species were proposed: CK6A, type II and CK7, type II [10]. Likewise, for *m/z* 1220.7, two possible peptide species were proposed: CK17, type I; and CK19, type I [10]. Spectra from identifications are provided in Figure S2.

## 3. Discussion

Reliable NSCLC subtyping is paramount for therapy stratification. In the current study, we analyzed a TMA consisting of 1 mm duplicate cores from 939 NSCLC FFPE tissue specimens and demonstrated that reliable entity subtyping is feasible by MSI on very limited tissue material.

Our data confirm that MSI measurements of multiple TMA sections are comparable if standardized protocols are implemented [12,13].

The application of a novel high-speed mass spectrometer resulted in a significantly reduced analysis time of less than one hour compared to the previously used instrumentation [9]. Although not analyzed in detail in the present study, overall turnaround time is comparable and may even be favorable compared to IHC, which is the current gold standard.

The classification of MSI data has been mainly performed using LDA and SVM [14,15,16,17,18,19,20]. An RF classification algorithm has previously been applied to classify MSI data but not on a large clinically relevant sample cohort [21,22]. Compared to other machine learning algorithms, classification based on decision trees has the advantage of being non-parametric. Moreover, they are able to detect multivariate interacting effects between variables and have good scalability [23]. In our study, the RF algorithm outperformed LDA and SVM in classifying a validation set based on training set data and may be more suitable than LDA or SVM to solve classification tasks using MSI data. However, this needs to be investigated in further studies.

The results obtained from MSI analysis seem plausible, as the gold standard IHC markers were closely correlated with the MSI classification results, as shown by t-SNE visualization. Interestingly, the classification accuracy with all variables was also stable with fewer variables towards an optimum of 91.2% before it rapidly decreased using less than 6 *m/z* peaks. We therefore conclude that classification based on a limited number of highly potent variables results in high classification accuracy and may outperform a larger number of variables.

The optimal sensitivity and specificity in the validation cohort were 92.9% and 89.3%, respectively, using MSI data. This result is comparable to the accuracy achieved with the currently recommended IHC markers, which has been reported to be 92%, but is less accurate than our previous MSI results [24]. This discrepancy may mainly be due to the increased sample size and precision in the current study. As regular staining may be performed after MSI, no tissue is lost during the analytical workup, which is a substantial benefit in small biopsy specimens, especially when remaining tissue material is needed for predictive molecular testing [25].

The identification of the most important *m/z* peaks for classification revealed proteins that are currently used in routine diagnostics and are well established in the classification of NSCLC such as CK5 and CK6. Interestingly, CK7 was also among the *m/z* values that were identified as relevant for the differentiation between ADC and SqCC. Although not currently recommended for the IHC differentiation of both entities due to low specificity [26], our results suggest that CK7 potentially adds value when used in a larger panel rather than as a single biomarker. In our study, 97% and 21% of ADC and SqCC samples, respectively, were positive immunohistochemically for CK7, which is well in line with the MSI results and with the reported frequencies in both tumors [27,28,29,30].

It is well known that cancer may induce a stromal reaction associated with the expression of specific collagen species [31]. The expression of collagen type I has been previously shown to be produced in NSCLC and fibroblasts [32] and was associated with EGFR tyrosine kinase inhibitor resistance in EGFR-mutated cancer cells [33]. Although collagen type I was among the *m/z* values that contributed most to the differentiation of ADC and SqCC, its biological role needs to be studied in more detail. As we investigated the samples at a resolution of 50 µm, we cannot fully exclude the possibility that, despite elaborate annotation of the tumor area, the measured collagen species are derived from stromal fibroblasts at the tumor–stromal border.

In a routine diagnostic scenario MSI could be performed on a single tissue section, stained by conventional hematoxylin and eosin after the analysis. Using this approach, the mass spectrometric data would be available in addition to the morphological data without additional tissue consumption.

## 4. Materials and Methods

### 4.1. Sample Selection

FFPE NSCLC specimens collected from 2002 to 2010 in the Thoracic Hospital Heidelberg at Heidelberg University were extracted from the archive of the Institute of Pathology, Heidelberg University, with the support of the tissue bank of the National Center for Tumor Diseases (NCT, #2591 and #2335). The tissues were used in accordance with the ethical regulations of the NCT tissue bank defined by the local ethics committee (ethics committee of the University of Heidelberg, #S-207/2005 and #S315/2020). Diagnoses were made according to the recommendations of the World Health Organization classification for lung cancer 2015 [34]. A cohort of 939 NSCLC patients, including ADC (*n* = 499) and SqCC (*n* = 440) patients, was selected. The results from conventional NSCLC markers for diagnostic subtyping—such as CK5/6, p40, Napsin-A, and TTF-1—were stained and published previously [24,35]. The patient characteristics and immunohistological staining results are summarized in Table 1. Typical IHC characteristics are outlined in Figure S3.

### 4.2. Workflow

**Figure 4:**
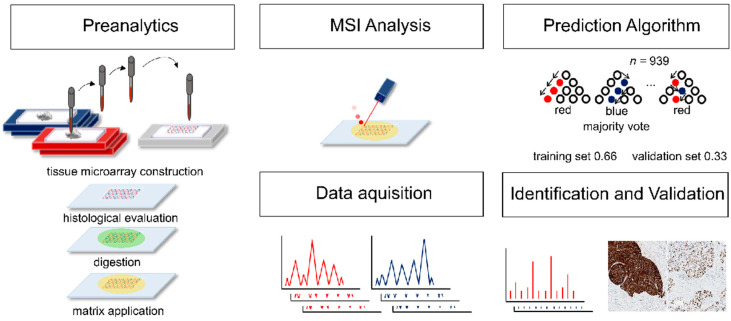
Workflow of mass spectrometry imaging analysis and data evaluation. Tissue microarrays were constructed and analyzed by mass spectrometry imaging. Samples were randomly assigned to a training set (66% of samples) and a test set (33% of samples). A prediction algorithm was created on the training set and validated on the test set. After data evaluation, the most important *m/z* values were identified by tandem mass spectrometry and validated using immunohistochemistry.

After cohort selection, TMAs were constructed as previously described [35,36,37], including duplicate 1 mm tissue cores for each case derived from representative, well preserved tumor areas. All TMAs were analyzed by MSI. Tumor regions were marked, and data from regions with high tumor cell content were extracted. Samples were randomly assigned to a training set and a test set (two-thirds and one-third of samples, respectively). A prediction algorithm was created on the training set and validated on the test set. The data were evaluated in detail to reveal *m/z* values that were most relevant for the classification. These *m/z* values were identified by tandem mass spectrometry (MS/MS) and validated using IHC. A summary of the workflow is depicted in Figure 4.

### 4.3. Matrix-Assisted Laser Desorption/Ionization Time-of-Flight Mass Spectrometry Imaging Analysis and Tandem Mass Spectrometry Analysis for Identification

Three-micrometer-thick sections of each specimen were cut and mounted onto conductive indium tin oxide-coated glass slides (Bruker Daltonik, Bremen, Germany) previously-coated with poly-L-lysine. The sample slides were processed for dewaxing with xylene (Fischer Scientific, Schwerte, Germany), rehydrated through graded ethanol washes (VWR, Darmstadt, Germany), and subjected to heat-induced antigen retrieval in deionized water at 95 °C for 30 min, as previously described [9]. For on-tissue digestion, trypsin solution was prepared in 800 µL of 20 mM ammonium bicarbonate (Sigma-Aldrich, Darmstadt, Germany) to a final concentration of 0.025 µg/µL and sprayed onto tissues with an automatic reagent sprayer (TM-sprayer, HTX Technologies, Chapel Hill, NC, USA). The spraying parameters were as follows: 8 passes, 30 °C temperature, 30 µL/min flow rate, 750 mm/min velocity, 2 mm track spacing, crisscross pattern, 10 psi pressure, 2 L/min gas flow rate, no drying time, and 40 mm nozzle height. The sections were subsequently incubated in a humidified chamber at 50 °C for 2 h. A solution of 10 mg/mL alpha-cyano-4-hydroxycinnamic acid matrix (Bruker Daltonik) in 70% acetonitrile (Honeywell Riedel-de Haen, Seelze, Germany)/1% trifluoroacetic acid (Millipore, Darmstadt, Germany) was then applied onto the digested sections using the same spraying device as above. The parameters for matrix spraying were as follows: 4 passes, 75 °C temperature, 120 µL/min flow rate, 120 cm/min velocity, 3 mm track spacing, HH pattern, 10 psi pressure, 2 L/min gas flow rate, no drying time, and 40 mm nozzle height.

MSI was performed using a mass spectrometer (rapifleX MALDI Tissuetyper, Bruker Daltonik) operated in reflectron mode with positive polarity for MSI analysis and in LIFT mode for MS/MS spectra acquisition. MSI runs were programmed using flexImaging version 5.0 (Bruker Daltonik). Each spectrum was automatically generated at a spatial resolution of 50 µm using flexControl version 4.0 (Bruker Daltonik) in the mass range of *m/z* 500–3200. Five hundred laser shots were acquired for each spectrum at 10 kHz. A peptide calibration standard mix including bradykinin, angiotensin II, angiotensin I, substance P, bombesin, ACTH clip 1-17, ACTH clip 18-39, and somatostatin 28 (Bruker Daltonik) was used for external calibration. Following MSI measurements, the matrix was removed by two washes in 100% methanol (Fischer Scientific) for 5 min each and stained with hematoxylin and eosin.

For in situ identification, the MS/MS spectra generated were submitted to the MASCOT server (Mascot v. 2.6.2.1), where a SwissProt database search (SwissProt_2018_09. Fasta; http://www.matrixscience.com/) was used to match tryptic peptide sequences to their respective intact proteins. The MS/MS spectrum search parameters included an MS tolerance of 200 ppm, MS/MS tolerance of ±0.5 Dalton, up to two missed cleavages, methionine oxidation, protein N-terminal acetylation, and proline oxidation as variable modifications.

### 4.4. Data Processing

Data processing and image visualization were performed using SCiLS Lab version 2018b (SCiLS, Bremen, Germany). Tumor areas were annotated by a thoracic pathologist (Mark Kriegsmann) in SCiLS Cloud (SCiLS; https://scils-cloud.de/) to ensure that only data from tumor containing areas and not from stroma was processed. During the annotation process necrotic areas and tissue regions with poor preservation were avoided. Baseline subtraction was performed using a convolution algorithm. Peak picking was performed manually to avoid artifactual peak selection. All monoisotopic peaks above baseline were picked. Spectra normalization was performed using the total ion count algorithm. The mean intensities for each core and each peak from the resulting peaklist were exported and subsequently analyzed by R statistical software (v.3.6.0). To ensure that no background noise was selected for further analysis, we decided to take only peaks with an absolute intensity ≥1.

### 4.5. Immunohistochemical Validation

IHC staining was performed using antibodies against CK5 and CK6 (combined antibody CK5/6), CK7, TTF-1, Napsin-A, and p40 according to quality controlled and accredited protocols (ISO-9001, DIN 17020) to ensure diagnostic validity. Tissue sections were pretreated with an antigen retrieval buffer and stained using an automated device (Ventana Benchmark Ultra, Roche, Mannheim, Germany) as previously described [12]. A summary of the antibody characteristics and staining protocols is provided in Table S2.

### 4.6. Statistical Analysis

Statistical data analysis was performed using R statistical software (v. 3.6.0; www.r-project.org, v.3.4.2, Free Software Foundation) and RStudio (1.2.5033, Affero General Public License, Boston, MA, USA) with the ‘caret’, ‘kernlab’, ‘random forest’, ‘ggplot2’, and ‘Rtsne’ packages. RF, SVM and LDA were used for the regression and classification of lung tumors (ADC versus SqCC). The algorithms were trained on 2/3 of all randomly selected cases (training set). Classification was performed on one-third of the cohort (validation set), and the prediction accuracy was calculated based on the proportion of correctly classified cases within the validation set. The selection of *m/z* peaks that contribute most to the RF model classifying ADC and SqCC samples was based on the mean decrease in the Gini coefficient. The *m/z* peak intensities in the ADC and SqCC samples were visualized using box plots. t-SNE was performed to visualize and validate the classification model based on the selected *m/z* peaks with regard to tumor entity, TMA, and IHC markers (TTF-1, Napsin, CK5/6, and p40).

## 5. Conclusions

In summary, our study shows that the subtyping of NSCLC into ADC and SqCC by MSI is rapid and accurate with limited tissue material.

## Figures and Tables

**Table 1:** Patient characteristics and immunohistochemical staining characteristics.

Variables	ADC	SqCC
Overall, *n*	499	440
Sex, *n* (%)		
male	291 (58)	368 (84)
female	208 (42)	72 (16)
Age, median (min–max)	63 (30–89)	65 (38–83)
TNM, *n* (%)		
pT1	93 (19)	85 (19)
pT2	321 (64)	259 (59)
pT3	70 (14)	78 (18)
pT4	15 (3)	18 (4)
pN0	252 (51)	207 (47)
pN1	70 (14)	148 (34)
pN2	155 (31)	78 (18)
pN3	4 (1)	0 (0)
pNX	18 (3)	7 (2)
pM1	24 (5)	5 (1)
pMX	475 (95)	435 (99)
Stage, *n* (%)		
I	201 (40)	149 (34)
II	101 (20)	171 (39)
III	173 (35)	115 (26)
IV	24 (5)	5 (1)
Immunohistochemistry		
CK5/6	10 (2)	412 (94)
CK7	474 * (97)	91 ^#^ (21)
Napsin-A	369 (74)	5 (1)
p40	19 (4)	414 (94)
TTF-1	433 (87)	3 (1)

* *n* = 8, not available; ^#^ *n* = 11, not available. Abbreviations: ADC, adenocarcinoma; CK, cytokeratin; TNM, size/direct extent of the primary tumor, degree of spread to regional lymph nodes (pN0 = no lymph node metastases, pN1 = lymph node metastases to ipsilateral peribronchial and or hilar or intrapulmonal lymph nodes, pN2 = lymph node metastases to ipsilateral mediastinal and or subcarinal lymph nodes, pN3 = lymph node metastases to contralateral or supraclavicular lymph nodes, pNX = evaluation of lymph nodes was not possible), presence of distant metastasis (pM1 = distant metastases, pMX = no information on distant metastases)—according to WHO classification 2015; SqCC, squamous cell carcinoma; TTF-1, thyroid transcription factor 1.

**Table 2:** Accuracy of the classification algorithm with subsequent reduction in included *m/z* values.

RF Model	#1	#2	#3	#4	#5	#6	#7	#8	#9
Included variables (*m/z* values)	all *m/z* values (*n* = 195)	1410.7 810.4 1406.7 865.4 878.5 1234.7 1220.7 1104.6	1410.7 810.4 1406.7 865.4 878.5 1234.7 1220.7	1410.7 810.4 1406.7 865.4 878.5 1234.7	1410.7 810.4 1406.7 865.4 878.5	1410.7 810.4 1406.7 865.4	1410.7 810.4 1406.7	1410.7 810.4	1410.7
			**Model Metrics**						
Prediction accuracy (CI_95%_)	0.906 (0.868–0.935)	0.906 (0.868–0.935)	0.903 (0.865–0.9328)	0.912 (0.875–0.941)	0.887 (0.847–0.919)	0.884 (0.843–0.917)	0.887 (0.847–0.919)	0.726 (0.674–0.775)	0.676 (0.622–0.727)
Sensitivity	0.947	0.929	0.929	0.929	0.894	0.882	0.894	0.781	0.686
Specificity	0.859	0.879	0.873	0.893	0.879	0.886	0.879	0.664	0.664
PPV	0.884	0.897	0.892	0.908	0.894	0.898	0.894	0.725	0.699
NPV	0.934	0.916	0.916	0.917	0.879	0.868	0.879	0.728	0.651
**Validation Data Set: *n* =318 (ADC *n* = 169, SqCC *n* = 149)**									
**Misclassifications, *n* (%)**									
ADC misclassified as SqCC	9 (5)	12 (7)	12 (7)	12 (7)	18 (11)	20 (12)	18 (11)	37 (22)	53 (31)
SqCC misclassified as ADC	21 (14)	18 (12)	19 (13)	16 (11)	18 (12)	17 (11)	18 (12)	50 (34)	50 (34)
Overall misclassified	30 (9)	30 (9)	31 (10)	28 (9)	36 (11)	37 (12)	36 (11)	87 (27)	103 (32)

ADC, adenocarcinoma; NPV, negative predictive value; PPV, positive predictive value; RF, random forest; SqCC, squamous cell carcinoma.

## Supplementary Materials

The following are available online at https://www.mdpi.com/2072-6694/12/9/2704/s1, Figure S1: Classification accuracy and variable importance of different algorithms, Figure S2: Spectra from Identifications, Figure S3: Typical examples of the immunohistological staining characteristics of adenocarcinoma and squamous cell carcinoma, Table S1: MS/MS identification results, Table S2: Antibodies and staining conditions.
