# Supplementary material for: Mass Spectrometry Imaging for Reliable and Fast Classification of Non-Small Cell Lung Cancer Subtypes
Source: Cancers (Basel). 2020 Sep 21;12(9):2704. doi: 10.3390/cancers12092704 (PMC7564257; doi:10.3390/cancers12092704)
Supplement: Supplementary file 1 [file cancers-12-02704-s001.pdf]

cancers

Article

Mass Spectrometry Imaging for Reliable and Fast Classification of Non-small Cell Lung Cancer subtypes

Mark Kriegsmann, Christiane Zgorzelski, Rita Casadonte, Kristina Schwamborn, Thomas Muley, Hauke Winter, Martin Eichhorn, Florian Eichhorn, Arne Warth, Soeren-Oliver Deininger, Petros Christopoulos, Michael Thomas, Thomas Longerich, Albrecht Stenzinger, Wilko Weichert, Carsten Müller-Tidow, Jörg Kriegsmann, Peter Schirmacher and Katharina Kriegsmann

Supplementary Materials

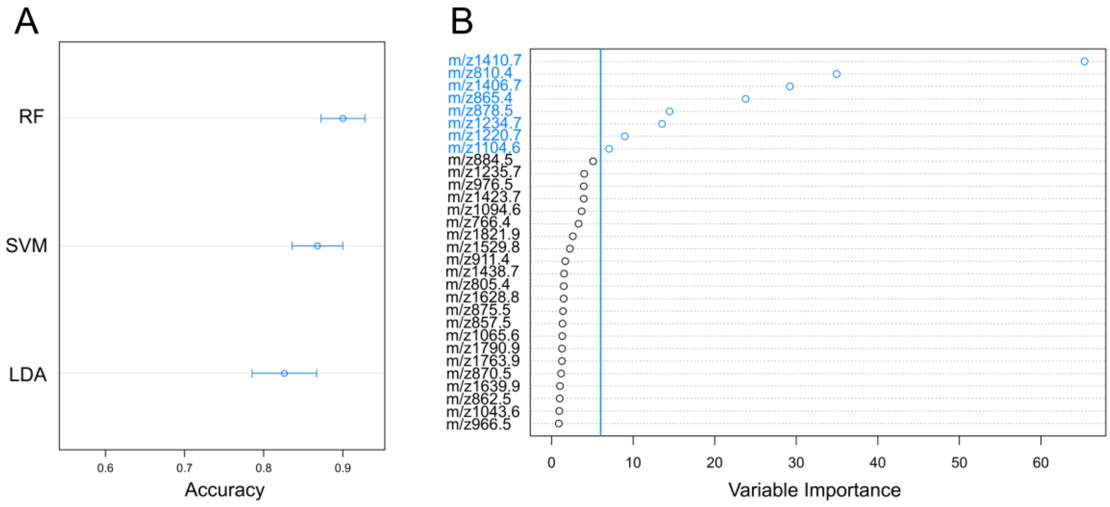

Figure S1. Classification accuracy and variable importance of different algorithms. Among the evaluated classification algorithms—RF (random forest), SVM (support vector machine) and LDA (linear discriminant analysis)—RF showed the highest level of accuracy in discriminating between ADC and SqCC samples (**A**). *m/z* peaks were ranked by variable importance to contribute to the RF classification algorithm. The 8 most important *m/z* peaks were selected for further evaluation (**B**).

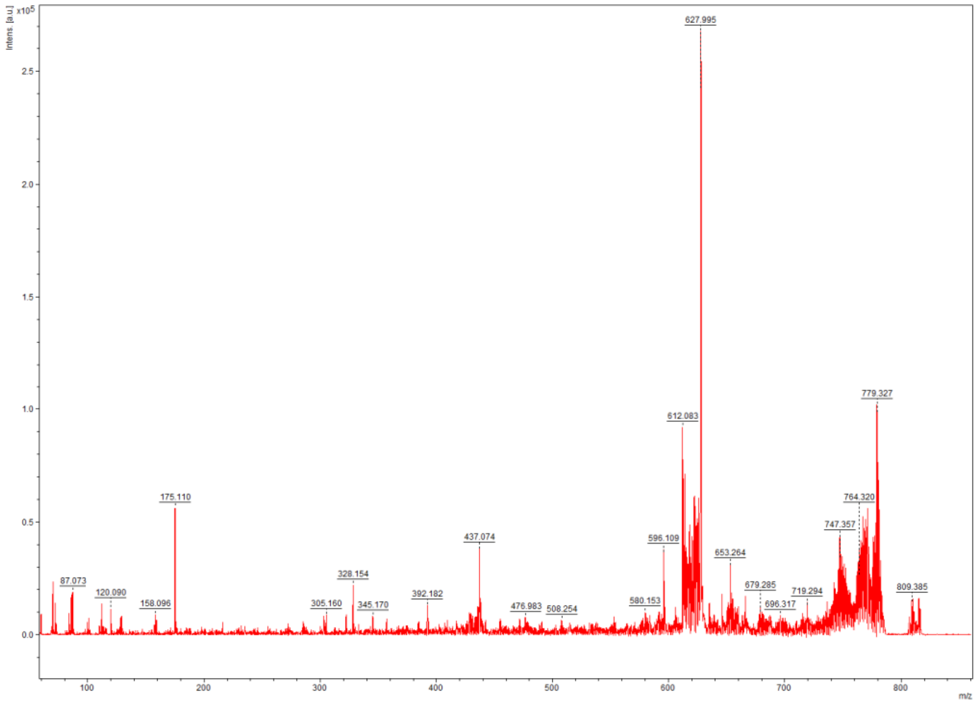
Intens. [a.u.]
x10^5
2.5
2.0
1.5
1.0
0.5
0.0
627.995
779.327
612.083
764.320
747.357
175.110
437.074
596.109
653.264
87.073
120.090
158.096
328.154
305.160
345.170
392.182
476.983
508.254
580.153
679.285
696.317
719.294
809.385
100
200
300
400
500
600
700
800
m/z

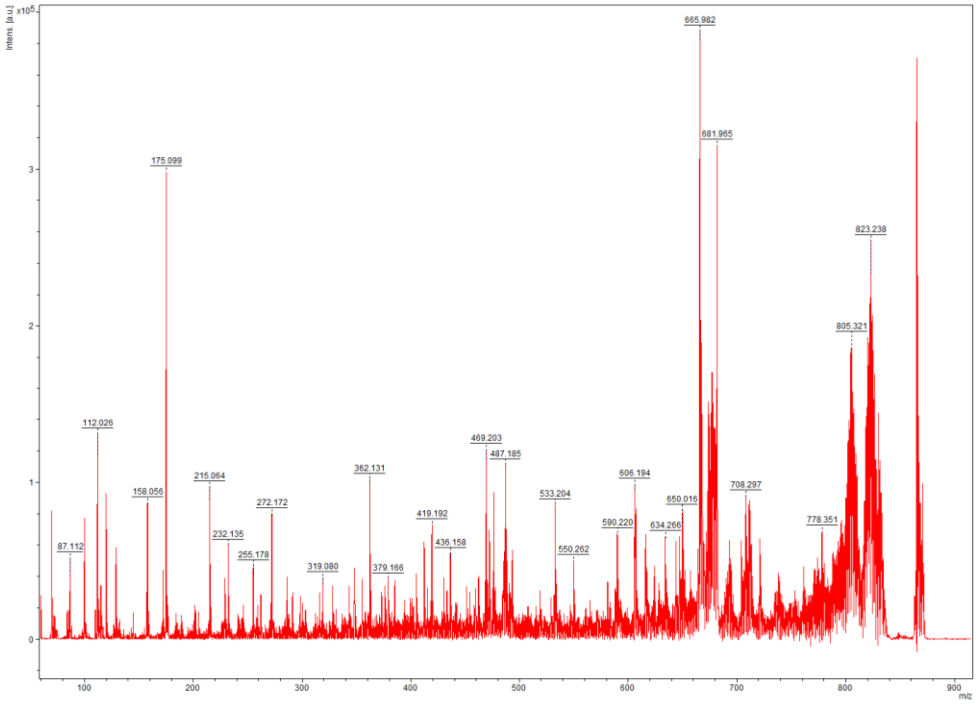
Intens. [a.u.]
x10^5
3
2
1
0
665.982
681.965
175.099
823.238
805.321
112.026
469.203
487.185
362.131
215.064
158.056
272.172
606.194
533.204
650.016
708.297
419.192
590.220
634.266
778.351
87.112
232.135
436.158
255.178
550.262
319.080
379.166
100
200
300
400
500
600
700
800
900
m/z

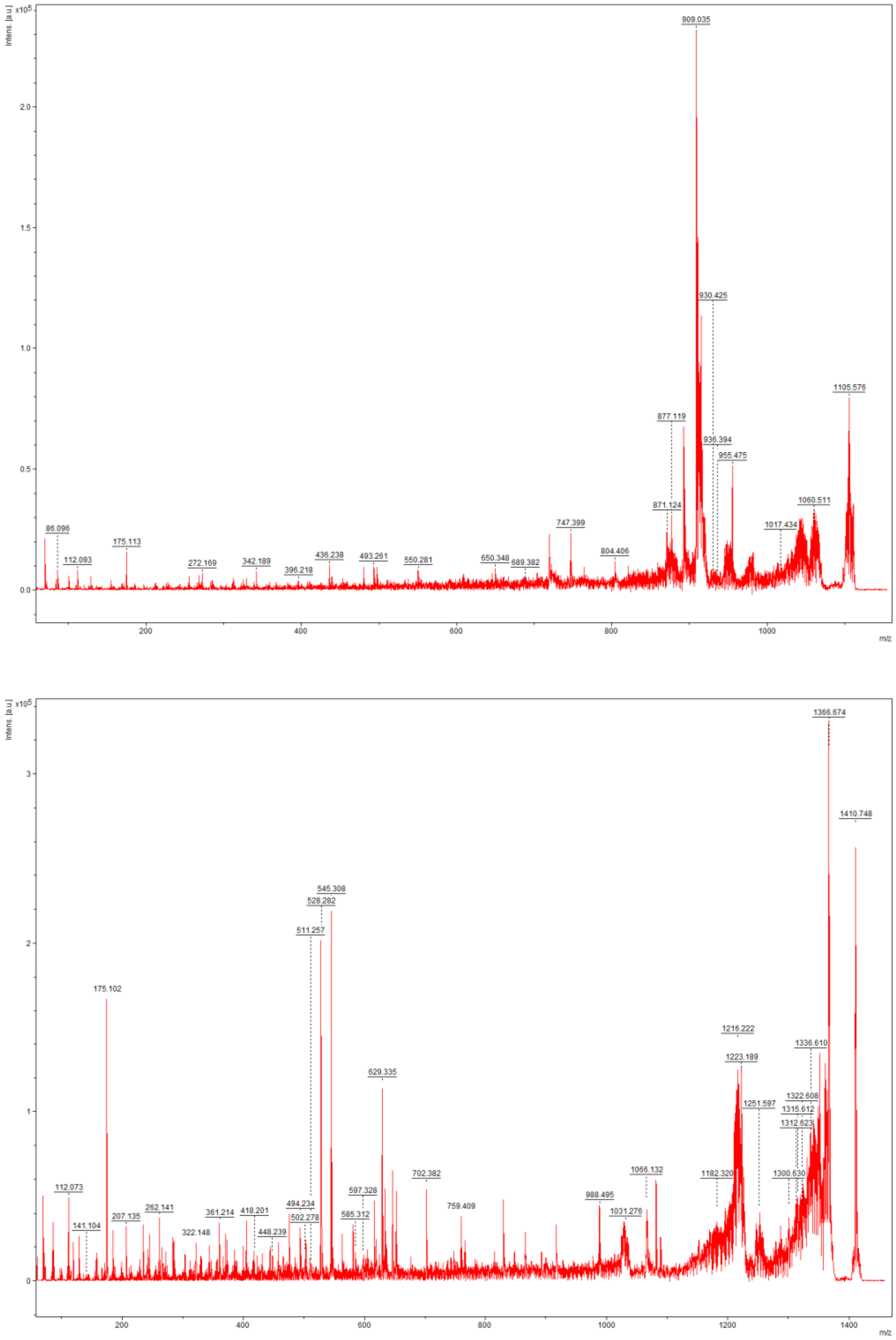

Figure S2. Spectra from Identifications.

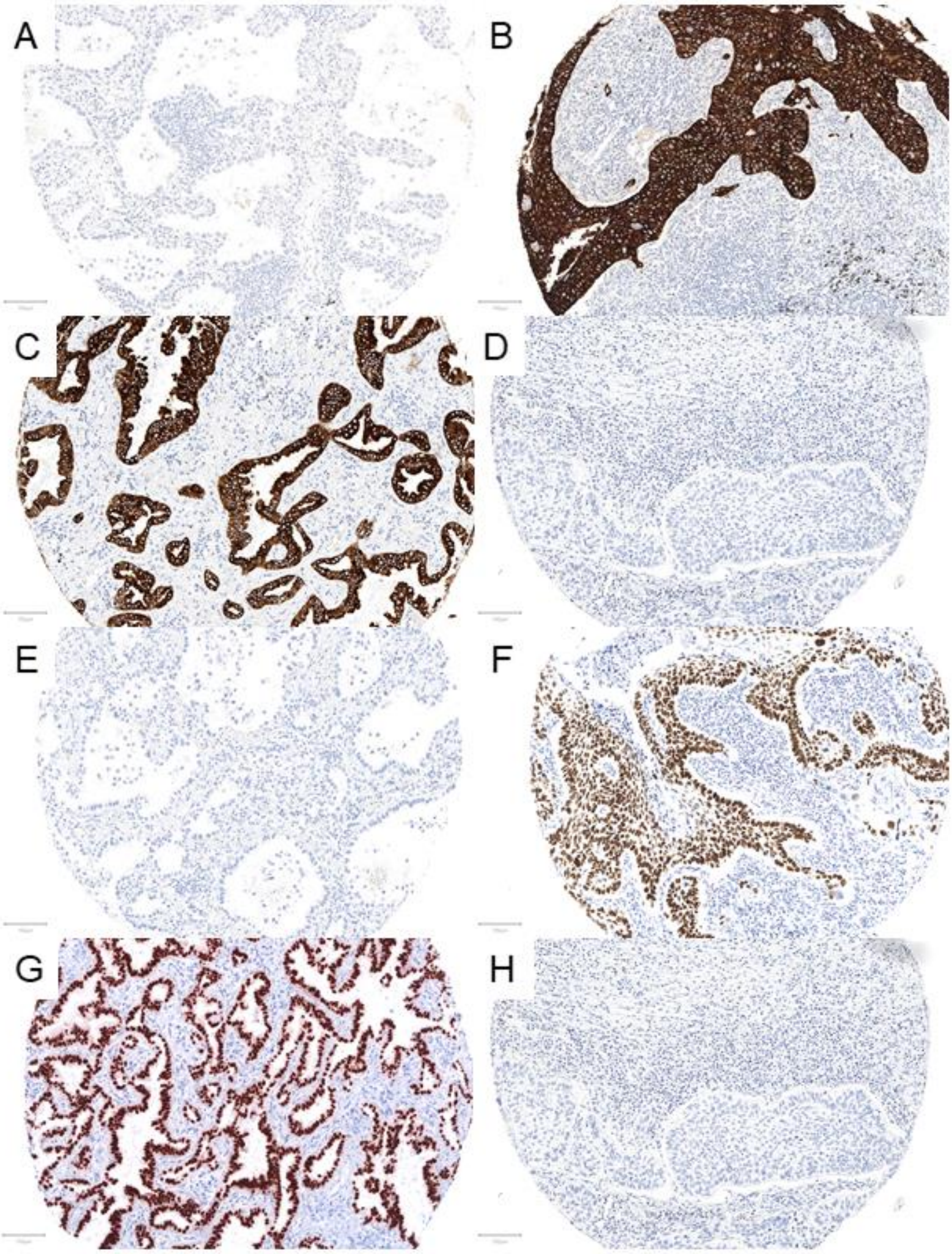

Figure S3. Typical examples of the immunohistological staining characteristics of adenocarcinoma and squamous cell carcinoma. Immunohistological staining of adenocarcinoma (left column) and squamous cell carcinoma (right column) are displayed. Adenocarcinomas are frequently positive for CK7 (**C**) and TTF-1 (**G**) and are negative for CK5/6 (**A**) and p40 (**E**). Squamous cell carcinoma is often positive for CK5/6 (**B**) and p40 (**F**) and is frequently negative for CK7 (**D**) and TTF-1 (**H**).

Table S1. MS/MS identification results.

Observed *m/z*	Mr (expt) *m/z*	Mr (calc) *m/z*	Mass Error (ppm)	Mascot Score	Peptide Sequence	Protein Name	Variable Modifications
**Identified in this study**							
810.4	809.3	809.4	−6.7	64	R.QSSVSFR.S	Keratin 5, type II	Acetyl (Protein N-term), Oxidation (M)
865.3	864.3	864.3	9.1	47	R.SGGGGGG GFGR.V	Keratin 5, type II	Acetyl (Protein N-term), Oxidation (M)
1104.5	1103.5	1103.5	−10.5	69	R.SAYGGPV GAGIR.E	Keratin 7, type II	Acetyl (Protein N-term), Oxidation (HW), Oxidation (M), Oxidation (P)
1410.7	1409.7	1409.7	26.7	42	R.SFSTASAIT PSVSR.T	Keratin 5, type II	Acetyl (Protein N-term), Oxidation (M)
**Identified in the literature**							
1220.6<br>1222.6 [10]	1221.6	1221.6	−2.95	55	R.TKFETEQA LR.M	Keratin 19, type I	
1222.6 [10]	1221.6	1221.6	−20.45	40	R.LEQEIATY R.R	Keratin 17, type I	
1234.6<br>1235.6[#]			−11.37	36	TGHPGTVGP AGIR	Collagen alpha-2(I) chain	
1406.7<br>1407.7 [11]	1406.7	1406.7	−1.01	34	K.ADTLTDEI NFLR.A	Keratin 6A, type II	
1406.7 [11]	1405.7	1405.7	2.63	61	M.SIHFSSPVF TSR.S + Acetyl (Protein N-term)	Keratin 7, type II	
**Not identified**							
878.4							

Table S2. Antibodies and staining conditions.

Antibody	Company	Clone	Pretreatment	Buffer Incubation Time (min)	Antibody Incubation Time (min)	Dilution
CK5/6	Ventana	D5/16 B4	Tris/Borat/EDTA, pH 8.4	56	24	RTU
p40	Ventana	BC28	Tris/Borat/EDTA, pH 8.4	48	24	RTU
CK7	Ventana	SP52	Tris/Borat/EDTA, pH 8.4	32	24	RTU
Napsin-A	Novocastra	IP64	Tris/Borat/EDTA, pH 8.4	32	24	1:400
TTF-1	Novocastra	SPT24	Tris/Borat/EDTA, pH 8.4	56	24	1:100

CK, cytokeratin; RTU, ready to use; TTF-1, thyroid transcription factor-1.
